# A Cross-Sectional Study on Self-Perceived Health and Physical Activity Level in the Spanish Population

**DOI:** 10.3390/ijerph19095656

**Published:** 2022-05-06

**Authors:** Ángel Denche-Zamorano, María Mendoza-Muñoz, Jorge Carlos-Vivas, Laura Muñoz-Bermejo, Jorge Rojo-Ramos, Raquel Pastor-Cisneros, Frano Giakoni-Ramírez, Andrés Godoy-Cumillaf, Sabina Barrios-Fernandez

**Affiliations:** 1 Health Economy Motricity and Education (HEME), Faculty of Sport Sciences, University of Extremadura, 10003 Caceres, Spain; andeza04@alumnos.unex.es; 2 Research Group on Physical and Health Literacy and Health-Related Quality of Life (PHYQOL), Faculty of Sport Sciences, University of Extremadura, 10003 Caceres, Spain; 3 Departamento de Desporto e Saúde, Escola de Saúde e Desenvolvimento Humano, Universidade de Évora, 7004-516 Évora, Portugal; 4 Promoting a Healthy Society Research Group (PHeSO), Faculty of Sport Sciences, University of Extremadura, 10003 Caceres, Spain; raquelpc@unex.es; 5 Social Impact and Innovation in Health (InHEALTH), University of Extremadura, 10003 Caceres, Spain; lauramunoz@unex.es (L.M.-B.); jorgerr@unex.es (J.R.-R.); sabinabarrios@unex.es (S.B.-F.); 6 Faculty of Education and Social Sciences, Universidad Andres Bello, Las Condes, Santiago 7550000, Chile; frano.giakoni@unab.cl; 7 Grupo de Investigación en Educación Física, Salud y Calidad de Vida, Facultad de Educación, Universidad Autónoma de Chile, Temuco 4780000, Chile; andres.godoy@uautonoma.cl

**Keywords:** physical activity, sedentary behaviour, health, self-perceived health, health surveys

## Abstract

One-third of the Spanish population over 15 years of age did not achieve a reasonable amount of physical activity (PA) before the COVID-19 pandemic. We aim to analyse the associations between the PA level (PAL) and self-perceived health (SPH) in the Spanish population aged 15–69 years during the pre-pandemic period. A cross-sectional study was conducted using the Spanish National Health Survey 2017 (ENSE 2017) data, with 17,777 participants. We carried out a descriptive analysis, analysed intergroup differences with non-parametric statistical tests, and calculated the Odds Ratio (OR) and Relative Risk (RR) of having a negative SPH according to PAL. In addition, correlations between PAL and SPH were studied, finding associations between them (*p* < 0.001). Thus, performing moderate and intense PA was related to better SPH than just walking or inactive. Weak and moderate correlations were found between PAL and SPH (*p* < 0.001). We also found high ORs and RRs of negative SPH as PAL decreased. Moderate and intense PA were related to positive SPH, while the risk of negative perception in inactive people was higher.

## 1. Introduction

By 2011, it was estimated that one in five people worldwide performed insufficient PA [1]. This prevalence has been increasing in recent years; before the COVID-19 pandemic, a third of the global population over 15 years performed insufficient physical activity (PA), according to the World Health Organization (WHO) and other organisations’ recommendations [2,3,4]. One of the goals established by the WHO in its Global Action Plan on Physical Activity 2018–2030 [5] is to reduce physical inactivity by 10% in 2025 and by 15% in 2030. Thus, in the last guideline published by this organisation before the COVID-19 pandemic, The WHO Guidelines on Physical Activity and Sedentary Behavior [6], it was recommended to engage in 150–300 min of moderate PA per week, 75–150 min at a vigorous intensity, or an equivalent combination of both [6]. In Spain, these recommendations were not reached by more than a third of the population in 2017, with a higher insufficient PA in women (37% women; 33.5% men), according to the report published by the Spanish Ministry of Health, Consumer Affairs and Social Welfare General Technical Secretary [7].

PA, defined as any bodily movement produced by skeletal muscles that require energy expenditure, is associated with multiple health benefits [8]. PA is inversely correlated with weight gain, decreased obesity, and associated comorbidities [9], and reduced risk of suffering some types of cancer, including breast, colon, liver, and lung cancer [10,11]. Other findings suggest an association with lower incidence and/or better glycemic control in type II diabetes and metabolic syndrome [12]. In addition, there is evidence of an association with improved symptoms related to mental illness [13,14], reduced pain [15], and better health-related quality of life [16]. In contrast, physical inactivity, which represents the non-achievement of physical activity guidelines [8], is associated with a deterioration in health indicators such as the increased prevalence of overweight and obesity [9], is related to cardiovascular diseases [17], some forms of cancers [18], increased prevalence of pain [19], and increased symptomatology in mental illnesses [20], thus influencing individuals’ perception of health.

Self-Perceived Health (SPH) “represents a summary statement about how numerous aspects of health, both subjective and objective, are combined within the perceptual framework of the individual respondent”, being a powerful indicator of people’s health status [21]. Thus, SPH is associated with age, sex, educational level, marital status, socioeconomic status, social environment, support, and behaviours that impact physical and mental health [22]. Furthermore, associations have been found between SPH and objective health, being a reliable predictor of people’s health status [23], as it integrates objective knowledge of potential medical conditions with the interpretation of the individual’s physical and mental signs [24].

This study aims to analyse the potential associations between PA level (PAL) and self-perceived health (SPH) in the Spanish adult population in the last Spanish National Health Survey, the ENSE 2017, [25] before the COVID-19 pandemic, providing a frame of reference for research comparing with post-pandemic periods. Data will be examined in the general population and between ages and sexes. In addition, the odds ratios (OR) and relative risks (RR) of negative health perception, according to the population’s PAL, will be presented. It was hypothesised that PAL would be positively correlated with SPH, so the population who perform moderate or vigorous-intensity PA will exhibit better SPH than those who only walked or were sedentary.

## 2. Materials and Methods

### 2.1. Study Desing

A descriptive correlational study was conducted using The Adult Questionnaire from the ENSE 2017 [25]. The Spanish Ministry of Health, Consumer Affairs and Social Welfare, together with the National Institute of Statistics, conducts the ENSE every five years to explore a variety of health-related factors in the Spanish population to plan and evaluate health policies. The ENSE 2017 was conducted between October 2016 and October 2017. After the random selection of participants, the Ministry sends a letter to the participants, providing information on the goals and characteristics of the study, explaining its confidential nature, requesting their voluntary collaboration, and warning them of the arrival of the accredited interviewers.

### 2.2. Ethical Concerns

Following the 2016/679 Regulation from the European Parliament and the Council of 27 April 2016 on the protection of individuals in the field of personal data processing and on the free movement of personal data and derogating Directive 95/46/EC (General Data Protection Regulation) [26], public use files are not considered confidential, so there is no requirement to apply data protection principles to anonymised information or the approval of accredited ethics committees, even for statistical or research purposes.

### 2.3. Participants

**Figure 1:**
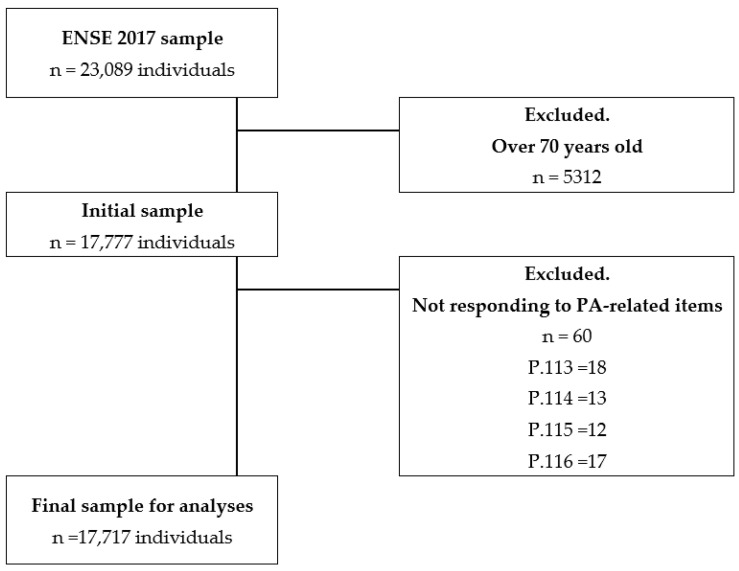
Sample exclusion criteria.

In order to conduct the ENSE 2017, 23,089 people (10,595 men and 12,494 women) residing in Spain and aged 15 and over were selected using a random stratified 3-phase sampling system [27]. To configure the final sample for this research, those over 70 years (5312 individuals) were excluded as they were not asked about their PA level. Thus, the sample consisted of 17,777 people, 8529 men and 9248 women, aged between 15 and 69, who were interviewed using the Adult Questionnaire [28]. Nevertheless, 60 additional participants were excluded for non-response to Q.113 and Q.114 items related to intense and moderate PA frequency and duration. Finally, 17,717 people were included in the analyses, as shown in Figure 1.

### 2.4. Measures and Variables

After the extraction of ENSE 2017 microdata, two types of variables were used:

- Extracted variables: Related to the items “Age”, “Sex” (male and female), G21 (self-perceived health, with possible answers: very bad, bad, fair, good, good, very good, do not know, or no response (NS/NC)), Q.113 (weekly frequency of intense PA), Q.114 (average duration of intense PA performed), Q.115 (weekly frequency of moderate PA), Q.116 (average duration of moderate PA performed), and Q.117 (time spent walking in the last seven days).
- Elaborated variables:
  - Age groups: Taking data from the item “Age”, the new variable Age groups were created: young people (15–35 years), young adults (35–49 years), older adults (50–64 years), and older adults (65–69 years) [29].
  - Physical Activity Index (PAI): With data from the PA-related items, the procedure followed in previous studies were used to build the PAI variable [30,31], using an adaptation of the Nes et al. Physical Activity Index [32], with values between 0 and 67.5 using the items Q.113, Q.114, Q.115, and Q.116. These corresponded to questions on the PA of the participants using the Spanish version of the International Physical Activity Questionnaire (IPAQ) [33]. The PAI was calculated using the following formula: (intensity factor for intense activity × frequency factor for intense activity × duration factor for in-tense activity) + (intensity factor for moderate activity × frequency factor for moderate activity × duration factor for moderate activity). Previously, different factors had been assigned to the participants’ answers to ENSE 2017 [28] items Q.113, Q.114, Q.115, and Q.116. The intensity factor was 10 for intense activity and 5 for moderate activity, questions Q.114 and Q.116. Frequency factor: to the answers given by the participants to items Q.114 (“How many days did you do intense PA?”) and Q.116 (“How many days did you do moderate PA?”), the following factors were applied: 0 for never, 1 for one day a week, 2 for 2–3 days a week, and 3 for more than three days a week [26]. Duration factor: to the answers given by the participants to items Q.115 (“How much time did you spend in total on intense PA?”) and Q.116 (“How much time did you spend in total on moderate PA?”), factor 1 (<30 min) and 1.5 (>30 min) were applied.
  - Physical Activity Level (PAL): Participants were grouped according to their PAI. In addition, item Q.117 regarding the time spent walking during the last seven days was used to discriminate between walkers and inactive people within PAI = 0. A total of 6 levels were established. On the one hand, there were two levels corresponding to individuals with PAI = 0: “Inactive”, those who did not perform moderate or intense PA for more than 10 min any day of the week and those who responded with “no day more than 10 min consecutively”, and “Walkers”, who responded with “one or more days, more than 10 min consecutively”. On the other hand, there were 4 PA levels for people with PAI > 0. These last four levels correspond to the 75th, 90th, 95th, and higher percentiles concerning all participants’ PAI considering the following: “Low” for persons with a PAI between 1 and 5, 75th percentile; “Medium” for PAI between 16a and 30, 90th percentile; “High”, PAI between 31 and 45, 95th percentile; and “Very high”, PAI > 45, values higher than the 95th percentile.

### 2.5. Statistical Analysis

Statistical analysis was conducted with the Statistical Package for the Social Sciences software (SPSS, Version 25, IBM SPSS, Armonk, NY, USA). The distribution followed by the data of the variables of interest was studied using the Kolmogorov–Smirnov test. After performing the Kolmogorov–Smirnov test, we could not assume that variables followed a normal distribution, so median, interquartile range (continuous variables), and absolute and relative frequencies (categorical variables) were taken as reference, and non-parametric tests were used. A descriptive analysis was carried out to characterise the sample. Potential intergroup differences were explored: the Mann–Whitney *U* test for continuous variables, the Chi-Square statistic, and a Z-test to compare proportions for the ordinal ones. According to the PAL, odds ratios (OR) and relative risks (RR) of perceiving negative health were also calculated. A correlation study was performed using Spearman’s rho between variables of interest. Finally, stepwise regression analysis to predict self-perceived health was performed considering age group, sex, and physical activity level as dependent variables. A significance level lower than 0.001 was required to introduce a new variable into each prediction model. The overall predictive power was evaluated by adjusted R^2^.

## 3. Results

In Table 1, several aspects are displayed. No intergroup differences in age were found between sexes (*p* = 0.295), nor dependency associations between sex and age group (*p* = 0.260). Overall, 74% perceived their health status as “Good” (52%) or “Very good” (22%), with a higher sex-dependent association in men than in women (*p* < 0.001). Men’s proportion with “Good/Very good” SPH was higher than women’s by 6 points (77.1% vs. 71.1%, with significant differences in SPH between sexes (*p* < 0.05). However, 59.8% of the population reported not performing moderate or intense PA. Furthermore, one of seven reported not performing any PA, not even walking, finding dependency relations between PAL and sex (*p* < 0.001). A higher proportion of women (50.7% vs. 39.9%, *p* < 0.05) stated that they only walked. In contrast, the men’s proportion with “Medium” to “Very high” PA was higher than in women (33% vs. 20.4%), with significant differences between sexes in all PA (*p* < 0.05), except for inactive ones.

Table 2 reveals how PAL relates to age, with a dependency relation with the age group (*p* < 0.001). A decrease in moderate or intense PA was found while the age increases: 4.6% of the population aged 65–69 years had a “High/Very high” PA, compared to 20.9% under 35 years. In contrast, there was an increase in the people proportions which only walked, from 35.8% of 35 years to 58% over 65 years.

PAL is also related to SPH, with a dependency relation both in the general population and by sex (*p* < 0.001). In the general population, the highest “Bad/Very bad” SPH proportions were found in the “Inactive” (15.6%) and “Walkers” (7.1%) groups compared to “Medium to Very high” levels (less than 3%). The highest “Good/Very good” health proportions were found in the “High” (88.2%) and “Very high” (86.9%) levels, compared to 58.6% proportion in the “Inactive” and 69.9% in “Walkers” groups. By sex, women (16.4%) and men (14.7%) in the “Inactive” group also had the highest prevalence of “Bad/Very bad” SPH and the lowest prevalence of “Good/Very good” health (Table 3).

**Figure 2:**
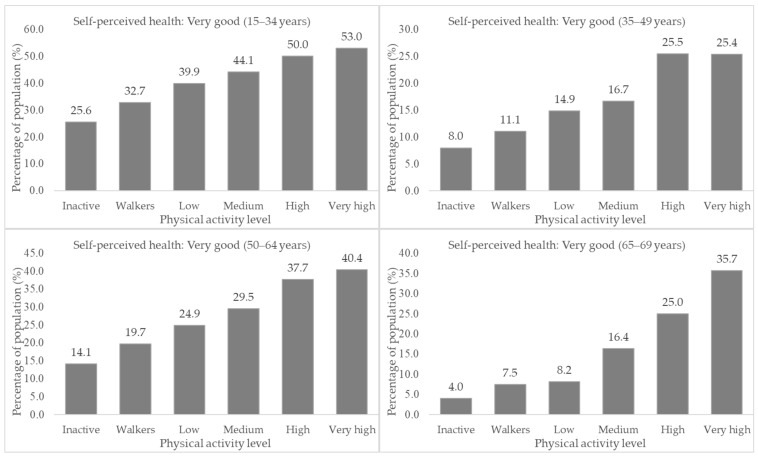
Associations between the level of physical activity and self-perceived health by age groups in the Spanish population through the ENSE 2017.

Table S1 (Supplementary Material) shows that the same dependency relations between the highest prevalence of “Very good” SPH were found in the groups with the highest PAL (Figure 2).

In Table 4, higher ORs and RRs of perceiving health as “Very poor” were found in the “Inactive” and “Walking” groups, while the lowest risk was found in the “High” group. For the “Poor” and “Fair” health states, ORs and RRs were also found to be higher for the lowest PAL compared to the highest (Table 4).

Finally, Table 5 reveals moderate correlations between age and SPH and weak ones between the PA level, age, and SPH.

A stepwise regression analysis to predict SPH was performed considering age group, sex and PAL as dependent variables. A significance level lower than 0.001 was required to introduce a new variable into each prediction model. The overall predictive power was evaluated by adjusted R^2^. The linear regression model presented a coefficient of determination R^2^ = 11.8%, positively explained by SPH (Constant: β = 1.778, t = 85.4, *p* < 0.001; Age Group: β = 0.227, t = 32.1, *p* < 0.001; PAL: β = −0.127, t = −27.4, *p* < 0.001; Gender: β = 0.060, t = 4.9, *p* < 0.001).

## 4. Discussion

### 4.1. Main Findings and Theoretical Implications

The main purpose of this research was to examine the association between PAL and SPH in the Spanish population between 15 and 69 years. Thus, being “Inactive” could have a negative impact on SPH compared to people who “Walk” or those who perform moderate or vigorous PA. The risk of perceiving a negative health status is elevated in “Inactive” individuals compared to those who walk and completed moderate or vigorous PA. Although being a “Walker” reduces the risk of negative SPH, the risk is higher concerning those engaged in higher-intensity PA. These findings have been found when classifying the population by sex and different age groups. No significant differences neither in age nor associations between sex and age group were found; it was assumed that the differences found between sexes in the rest of the analyses were not conditioned by age.

SPH was better in men than women, with a dependency ratio between SPH and sex. Higher proportions of people with a negative SPH were found in women than in men. This has been observed in other studies in Spain and other countries [34,35]. Women had a worse HPS than men for greater concern for their health [36], cultural, physiological, or biological issues [37]. Individuals in the “Walker” group had lower proportions of negative SPH states than the “Inactive” ones. This is in line with other studies that reported improvements when walking in different health indicators: decreased blood pressure, total cholesterol, BMI, resting heart rate, increased aerobic capacity, and improvement in depressive or anxiety symptoms [38,39,40]. Despite this, introducing moderate or vigorous intensities of PA produces more significant improvements [41,42,43].

Furthermore, most of the Spanish population was not physically active or did not practice moderate or vigorous PA enough to comply with the recommendations: three out of five persons (59.8%) reported not having practised moderate or vigorous PA during the week before the survey. Moreover, PAL was related to sex, and women’s proportion of not performing moderate or vigorous PA reached 65.4%, 11.7 points higher than men’s (53.7%). These findings are worrying compared to those found in other studies. For example, in works performed with 1.6 and 1.9 million people across 146 and 168 countries, a worldwide trend towards a prevalence of insufficient PA (27.5%), confirming the gap between men and women (23.4% vs. 31.7%) [3,4]. Given that SPH is related to PAL, this lower PAL in women could be one of the causes explaining the lower SPH found in women, with a 6% difference in the proportion of women with “Good/Very good” SPH. Among those who did not engage in moderate or vigorous PA, 45.5% at least walked for more than 10 min at a time, a minimum of 1 day a week. In this case, the women’s proportion was 10.8 percentage points higher than men’s, as found in other countries [38]. Women would prefer walking more than men instead of moderate or vigorous PA. The reasons for this must be still clarified. However, it is expected to be multi-causal, including different ways of understanding PA and obtaining pleasure, social, educational, economic issues, availability of vehicles for transport, lack of support, etc. [44,45,46].

In addition, associations were found between PAL and age, with a PAL decrease when increasing age. Other studies have documented 40–80% decreases in PA with growing age, related to muscle loss, the onset of metabolic disorders and other diseases [47]. In our study, the age group with the highest proportion of people performing moderate or vigorous PA was the young group, with 52%. However, up to 48% of Spanish young people did not perform at least moderate-intensity PA. The data were worse as age increased, reaching 73.5% of people who did not perform moderate or vigorous PA in the 65–69 age group. Only one in four people performed moderate or vigorous intensities PA in this population. However, up to 58% of the elderly did walk at least one day a week for more than 10 min at a time, 22.2% more than the younger proportion. People aged 65–69 had a greater preference for walking activities than moderate or intense PA. Some research has found these preferences among older adults and older people for slower-paced PA than younger people [47,48].

In the same way, SPH also decreased with age; these findings were shared with other papers [49]. Higher PAL was associated with a lower proportion of people with “Poor or Very poor” SPH. At the other extreme, “Very good” SPH went from 13.1% in sedentary people to 17.1% in people who only walked. The relationship between PAL and SPH found in this study reinforces the findings of other studies conducted in Spanish with youth and adolescents [50] or adult populations in other countries [51,52], confirming that PA influences health perception throughout the life cycle. Whether in the general population or across sexes or age groups, walking was associated with improved SPH than a sedentary lifestyle, given the multiple demonstrated health benefits [50,51,52]. Although walking is associated with a higher proportion of people with better SPH in the general population and across age groups, moderate or vigorous PA would be more beneficial even in older people (65–69 years) [42,43]. Following the above, the ORs and RRs of negative SPH were higher in population groups with lower PA. As an example, the probability of having a “Very bad” SPH was more than three times higher in “Inactive” than in “Walkers” and ten times higher concerning the highest PA levels when including moderate or intense PA. There is considerable scientific evidence to support our findings regarding the relationship between PAL and SPH [53,54,55] so that a higher PAL results in a lower risk of worse SPH.

The world has faced a pandemic that has led to, among other effects, social isolation of the population, affecting their lifestyle, PA [56], and SPH [57]. Numerous studies have shown that PA decreased during the pandemic [56,58,59,60] and that SPH worsened [61,62]. In line with our findings, studies in the pandemic showed positive associations between increased PAL and physical health and inverse associations between sedentary behaviour and physical and mental health outcomes [63,64,65].

### 4.2. Practical Implications

As found in different studies, the COVID-19 pandemic had negatively impacted SPH [66,67,68,69] and PAL [70,71,72,73]. Therefore, the significance of this study lies in the analysis of the associations between PAL and SPH in the Spanish adult population during the last period before the COVID-19 pandemic, serving as a frame of reference for future research analysing pre- and post-pandemic periods. Moreover, these results may support decision-making in creating strategic actions to promote active lifestyles and the practice of PA to improve the SPH of the population throughout the life cycle since PA could act in a preventive way to preserve the population’s SPH. Thus, a higher PAL is related to a better SPH, which could imply a lower psychological discomfort and an improvement in the physical and mental health of the population. Therefore, PAL and SPH could be considered important determinants of well-being and a psychosocial protective factor against the consequences of physical inactivity and low SPH during pandemic periods. These actions should have a gender perspective, as women have lower PALs, influencing their SPH [44,45].

It would be advisable for physical educators, health professionals, governments, or stakeholders to promote PA programs for all ages, including moderate or vigorous PA, to increase the population’s SPH. If this is not feasible, walking programs should be encouraged, as they are beneficial [42,43,74], although minor, especially in older people. Taking advantage of this willingness of older people to walk, programs to increase PA in this population group should be based on walking, especially in natural areas [75], to improve adherence and include moderate and vigorous activities to optimise results in health status indicators, whenever possible; not forgetting that play also involves movement and PA so that playfulness alternatives may also be considered because of its benefits in SPH and other areas [76].

### 4.3. Limitations and Future Lines

However, one of the study’s limitations is that differences between moderate and intense PA were not analysed, which should be considered in future research. Other limitations include the lack of participants’ medical history, PA objective and physiological data, and a follow-up. It would be advisable to carry out a 24-h compositional analysis, including devices to quantify PA or intensity or other measures that could overcome the limitations in this type of study based on surveys and the participants’ subjective perceptions. In this sense, PA measurement through questionnaires has significant limitations, and it would be advisable to use devices that can objectively quantify PA in these types of national surveys. In addition, the introduction of METs calculation from the IPAQ questionnaire could improve the validity of the study so that if we relate different possibilities of quantifying PA with the SPH, it could be the basis for future research. This work is a cross-sectional study based on national health surveys, which is already an intrinsic limitation regarding the study design. For this reason, it would be interesting to carry out complementary research with research designs which would allow establishing causal relationships. Carrying out this same study longitudinally through public institutions such as the Ministry of Health and the inclusion of objective PA data would be an excellent opportunity to improve the quality of the information obtained in this type of survey.

## 5. Conclusions

PAL is positively related to SPH, both in men and women and in the different age groups of the Spanish population.

The population groups that walked or performed moderate or vigorous PA had lower proportions of people with negative SPH. Walking could be an effective alternative to reduce the population with negative SPH compared to remaining inactive. However, it would be insufficient to reduce the proportions and risks of having negative SPH.

Age is associated with less SPH and lower PAL. However, increasing the population’s PAL in all age groups could reduce the proportions of people with negative SPH.

Being Inactive leads to elevated ORs and RRs in the population prevalence of negative health perception compared to walking or higher intensity PA. Only walking presents high ORs and RRs in this prevalence concerning those who perform moderate or intense PA.

## Figures and Tables

**Table 1:** Socio-demographic characteristics, self-perceived health, and the level of physical activity in the Spanish population through the ENSE 2017.

Ages (Years)	Total = 17,777	Men = 8529	Women = 9248	*p*
Median (IQR)	47 (21)	47 (21)	47 (21)	0.295
Mean (SD)	45.8 (14.1)	45.7 (14.1)	46.0 (14.1)	
Age Group (years)	Total *n* (%)	Men *n* (%)	Women *n* (%)	p *
15–34	3894 (21.9)	1864 (21.9)	2030 (22.0)	0.260
35–49	6195 (34.8)	3007 (35.3)	3188 (34.5)	
50–64	5977 (33.6)	2874 (33.7)	3103 (33.6)	
65–69	1711 (9.6)	784 (9.2)	927 (10.0)	
Self-perceived health	Total = 17,777 *n* (%)	Men = 8529 *n* (%)	Women = 9248 *n* (%)	
Very bad	263 (1.5)	97 (1.1)	166 (1.8) ^b^	<0.001
Bad	896 (5.0)	392 (4.6)	504 (5.4) ^b^	
Fair	3462 (19.5)	1457 (17.1)	2005 (21.7) ^b^	
Good	9252 (52.0)	4584 (53.7)	4668 (50.5) ^b^	
Very good	3904 (22.0)	1999 (23.4)	1905 (20.6) ^b^	
PAL	Total = 17,717 *n* (%)	Men = 8489 *n* (%)	Women = 9228 *n* (%)	
Inactive	2533 (14.3)	1175 (13.8)	1358 (14.7)	<0.001
Walkers	8067 (45.5)	3390 (39.9)	4667 (50.7)) ^b^	
Low	2433 (13.7)	1120 (13.2)	1313 (14.2) ^b^	
Medium	2456 (13.9)	1341 (15.8)	1115 (12.1) ^b^	
High	1472 (8.3)	954 (11.2)	518 (5.6) ^b^	
Very high	756 (4.3)	509 (6.0)	247 (2.7) ^b^	

IQR: Interquartile range; *n*: participants; %: percentage; Physical Activity Index (PAI) only considers intense and moderate physical activity. Scores between 0 and 67.5; Inactive: PAI = 0; reports not walking at least one day a week for more than 10 min at a time; Walkers PAI = 0; reports walking at least one day a week for more than 10 min at a time; Low: PAI = 1–15; Medium: PAI = 16–30; High: PAI = 31–45; Very high: PAI > 45; *p*: *p*-value from Mann–Whitney U test; *p* *: *p*-value from Chi-square test; ^b^ Significant differences between the proportions of each level, according to sex in the z-test for independent ratios (*p* < 0.05).

**Table 2:** Association between physical activity level and age group in the Spanish population through the ENSE 2017.

PAL	15–34 Years *n* (%)	35–49 Years *n* (%)	50–64 Years *n* (%)	65–69 Years *n* (%)	*p*
Inactive (*n* = 2533)	472 (12.2)	935 (15.1)	878 (14.7)	248 (14.5)	<0.001
Walkers (*n* = 8067)	1385 (35.8)	2565 (41.5)	3124 (52.5)	993 (58.0)	
Low (*n* = 2433)	572 (14.8)	970 (15.7)	696 (11.7)	195 (11.4)	
Medium (*n* = 2456)	637 (16.4)	881 (14.3)	743 (12.5)	195 (11.4)	
High (*n* = 1472)	560 (14.5)	554 (9.0)	306 (5.1)	52 (3.0)	
Very high (*n* = 756)	247 (6.4)	272 (4.4)	209 (3.5)	28 (1.6)	
Total (*n* = 17717)	3873	6177	5956	1711	

*n*: participants; %: percentage; absolute and relative frequency data shown for individuals in age groups for each level of physical activity; PAI: Physical Activity Index; Inactive: PAI = 0; reports not walking at least one day a week for more than 10 min at a time; Walkers PAI = 0; reports walking at least one day a week for more than 10 min at a time; Low: PAI = 1–15; Medium: PAI = 16–30; High: PAI = 31–45; Very high: PAI > 45; *p*: *p*-value from Chi-square test.

**Table 3:** Associations between the level of physical activity and self-perceived health in the Spanish population through the ENSE 2017.

Self-Perceived Health: General Population						
PAL	Very bad *n* (%)	Bad *n* (%)	Fair *n* (%)	Good *n* (%)	Very Good *n* (%)	*p*
Inactive (*n* = 2533)	120 (4.7)	275 (10.9)	603 (23.8)	1202 (45.5)	333 (13.1)	<0.001
Walkers (*n* = 8067)	110 (1.4)	460 (5.7)	1861 (23.1)	4257 (52.8)	1379 (17.1)	
Low (*n* = 2433)	11 (0.5)	80 (3.3)	399 (16.4)	1353 (55.6)	590 (24.2)	
Medium (*n* = 2456)	12 (0.5)	44 (1.8)	363 (14.8)	1340 (54.6)	697 (28.4)	
High (*n* = 1472)	6 (0.4)	19 (1.3)	148 (10.1)	719 (48.8)	580 (39.4)	
Very high (*n* = 756)	4 (0.5)	17 (2.2)	78 (10.3)	353 (46.7)	304 (40.2)	
TOTAL (*n* = 17,717)	263	895	3452	9224	3883	
**Self-Perceived Health: Women**						
**PAL**	**Very bad** ***n*** **(%)**	**Bad** ***n*** **(%)**	**Fair** ***n*** **(%)**	**Good** ***n*** **(%)**	**Very Good** ***n*** **(%)**	
Inactive (*n* = 1358)	71 (5.2)	152 (11.2)	360 (26.5)	599 (44.1)	176 (13.0)	<0.001
Walkers (*n* = 4677)	80 (1.7)	258 (5.5)	1112 (23.8)	2402 (51.4)	825 (17.6)	
Low (*n* = 1313)	7 (0.5)	57 (4.3)	248 (18.9)	699 (53.2)	302 (23.0)	
Medium (*n* = 1115)	3 (0.3)	23 (2.1)	180 (16.1)	596 (53.5)	313 (28.1)	
High (*n* = 518)	4 (0.8)	5 (1.0)	62 (12.0)	257 (49.6)	190 (36.7)	
Very high (*n* = 247)	1 (0.4)	9 (3.6)	38 (15.4)	107 (43.3)	92 (37.2)	
TOTAL (*n* = 9228)	166	504	2000	4660	1898	
**Self-Perceived Health: Men**						
**PAL**	**Very bad** ***n*** **(%)**	**Bad** ***n*** **(%)**	**Fair** ***n*** **(%)**	**Good** ***n*** **(%)**	**Very Good** ***n*** **(%)**	
Inactive (*n* = 1175)	49 (4.2)	123 (10.5)	243 (20.7)	603 (51.3)	157 (13.4)	<0.001
Walkers (*n* = 3390)	30 (0.9)	202 (6.0)	749 (22.1)	1855 (54.7)	554 (16.3)	
Low (*n* = 1120)	4 (0.4)	23 (2.1)	151 (13.5)	654 (58.4)	288 (25.7)	
Medium (*n* = 1341)	9 (0.7)	21 (1.6)	183 (13.6)	744 (55.5)	384 (28.6)	
High (*n* = 954)	2 (0.2)	14 (1.5)	86 (9.0)	462 (48.4)	390 (40.9)	
Very high (*n* = 509)	3 (0.6)	8 (1.6)	40 (7.9)	246 (48.3)	212 (41.7)	
TOTAL (*n* = 8489)	97	391	1452	4564	1985	

*n*: participants; %: percentage; absolute and relative frequency data shown for individuals in age groups for each level of physical activity; PAI: Physical Activity Index; Inactive: PAI = 0; reports not walking at least one day a week for more than 10 min at a time; Walkers PAI = 0; reports walking at least one day a week for more than 10 min at a time; Low: PAI = 1–15; Medium: PAI = 16–30; High: PAI = 31–45; Very high: PAI > 45; *p*: *p*-value from Chi-square test.

**Table 4:** Probability and relative risk of perceiving health as very poor, poor, or fair, according to the physical activity level in the Spanish population through the ENSE 2017.

Self-Perceived Health: Very Bad						
PAL		OR	CI 95%	RR	CI 95%	*p*
Inactives	Walkers	3.60	2.77–4.68	3.48	2.69–4.48	<0.001
	Low	10.95	5.89–20.35	10.48	5.67–19.38	<0.001
	Medium	10.13	5.58–18.38	9.70	5.37–17.51	<0.001
	High	12.15	5.34–27.66	11.62	5.13–26.32	<0.001
	Very high	9.35	3.44–25.40	8.95	3.32–24.17	<0.001
Walkers	Low	3.04	1.64–5.67	3.02	1.63–5.60	<0.001
	Medium	2.82	1.55–5.12	2.79	1.54–5.06	<0.001
	High	3.38	1.48–7.70	3.35	1.47–7.60	<0.005
	Very high	2.60	0.96–7.07	2.58	0.95–6.97	0.052
**Self-Perceived Health: Bad**						
**PAL**		**OR**	**CI 95%**	**RR**	**CI 95%**	
Inactives	Walkers	2.01	1.72–2.36	1.90	1.65–2.20	<0.001
	Low	3.58	2.78–4.63	3.30	2.59–4.21	<0.001
	Medium	6.68	4.83–9.23	6.06	4.43–8.29	<0.001
	High	9.31	5.82–14.90	8.41	5.31–13.33	<0.001
	Very high	5.30	3.22–8.70	4.83	2.98–7.83	<0.001
Walkers	Low	1.78	1.40–2.27	1.73	1.37–2.19	<0.001
	Medium	3.32	2.43–4.53	3.18	2.34–4.32	<0.001
	High	4.62	2.91–7.34	4.42	2.80–6.97	<0.001
	Very high	2.63	1.61–4.29	2.54	1.57–4.09	<0.001
**Self-Perceived Health: Fair**						
**PAL**		**OR**	**CI 95%**	**RR**	**CI 95%**	
Inactives	Walkers	1.04	0.94–1.16	1.03	0.95–1.12	0.444
	Low	1.59	1.38–1.83	1.45	1.30–1.63	<0.001
	Medium	1.80	1.56–2.08	1.61	1.43–1.81	<0.001
	High	2.80	2.31–3.39	2.37	2.00–2.80	<0.001
	Very high	2.72	2.11–3.49	2.31	1.85–2.88	<0.001
Walkers	Low	1.53	1.36–1.72	1.41	1.28–1.55	<0.001
	Medium	1.73	1.53–1.96	1.56	1.41–1.73	<0.001
	High	2.68	2.25–3.20	2.29	1.96–2.69	<0.001
	Very high	2.61	2.05–3.31	2.24	1.81–2.77	<0.001

OR: Odd ratio; CI95%: Confidence Interval; RR: Relative risk; *p*: *p*-valor from Chi-square; PAI: Physical Activity Index; Inactive: PAI = 0; reports not walking at least one day a week for more than 10 min at a time; Walkers PAI = 0; reports walking at least one day a week for more than 10 min at a time; Low: PAI = 1–15; Medium: PAI = 16–30; High: PAI = 31–45; Very high: PAI > 45; *p*: *p*-value from Chi-square test.

**Table 5:** Associations between the physical activity level, age, and self-perceived health in the Spanish population through the ENSE 2017.

PAL						
Variables	Total		Men		Women	
	rho	*p*	rho	*p*	rho	*p*
Age	−0.161	<0.001	−0.229	<0.001	−0.092	<0.001
SPH	0.244	<0.001	0.269	<0.001	0.210	<0.001

PAL: physical activity level; SPH: self-perceived health; Rho: Spearman’s correlation coefficient with Bonferroni correction factor (*p* = 0.01).

## Data Availability

Datasets will be available under reasonable request.

## Supplementary Materials

The following supporting information can be downloaded at: https://www.mdpi.com/article/10.3390/ijerph19095656/s1, Table S1: Associations between the level of physical activity and self-perceived health by age groups in the Spanish population through the ENSE 2017.
