# Supplementary material for: A Cross-Sectional Study on Self-Perceived Health and Physical Activity Level in the Spanish Population
Source: Int J Environ Res Public Health. 2022 May 6;19(9):5656. doi: 10.3390/ijerph19095656 (PMC9104406; doi:10.3390/ijerph19095656)
Supplement: Supplementary file 1 [file ijerph-19-05656-s001.zip › ijerph-1682487-supplementary.pdf]

Table S1. Associations between the level of physical activity and self-perceived health by age groups in the Spanish population through the ENSE 2017.

Self-Perceived Health In Youth: 15-34 years						
PAL	Very bad n (%)	Bad n (%)	Fair n (%)	Good n (%)	Very Good n (%)	*P*
Inactive (n=472)	6 (1.3)	15 (3.2)	77 (16.3)	253 (53.6)	121 (25.6)	<0.001
Walkers (n=1385)	5 (0.4)	30 (2.2)	164 (11.8)	733 (52.9)	453 (32.7)	
Low (n=572)	2 (0.3)	6 (1.0)	57 (10.0)	279 (48.8)	228 (39.9)	
Medium (n=637)	0 (0.0)	3 (0.5)	57 (8.9)	296 (46.5)	281 (44.1)	
High (n=560)	1 (0.2)	7 (1.3)	21 (3.8)	251 (44.8)	280 (50.0)	
Very high (n=247)	0 (0.0)	2 (0.8)	13 (5.3)	101 (40.9)	131 (53.0)	
TOTAL (n=3873)	14	63	389	1913	1494	
**Self-Perceived Health In Young Adults: 35-49 years**						
PAL	Very bad n (%)	Bad n (%)	Fair n (%)	Good n (%)	Very Good n (%)	
Inactive (n=878)	62 (7.1)	137 (15.6)	248 (28.2)	361 (41.1)	70 (8.0)	<0.001
Walkers (n=3124)	69 (2.2)	245 (7.8)	841 (26.9)	1623 (52.0)	346 (11.1)	
Low (n=696)	4 (0.6)	37 (5.3)	150 (21.6)	401 (57.6)	104 (14.9)	
Medium (n=743)	6 (0.8)	22 (3.0)	158 (21.3)	433 (58.3)	124 (16.7)	
High (n=306)	5 (1.6)	5 (1.6)	55 (18.0)	163 (53.3)	78 (25.5)	
Very high (n=209)	3 (1.4)	12 (5.7)	36 (17.2)	105 (50.2)	53 (25.4)	
TOTAL (n=5956)	149	458	1488	3086	775	
**Self-Perceived Health In Older Adults: 50-64 years**						
PAL	Very bad n (%)	Bad n (%)	Fair n (%)	Good n (%)	Very Good n (%)	
Inactive (n=935)	33 (3.5)	69 (7.4)	193 (20.6)	508 (54.3)	132 (14.1)	<0.001
Walkers (n=2565)	24 (0.9)	112 (4.4)	527 (20.5)	1396 (54.4)	506 (19.7)	
Low (n=970)	5 (0.5)	20 (2.1)	147 (15.2)	556 (57.3)	242 (24.9)	
Medium (n=881)	6 (0.7)	16 (1.8)	100 (11.4)	499 (56.6)	260 (29.5)	
High (n=554)	0 (0.0)	5 (0.9)	61 (11.0)	279 (50.4)	209 (37.7)	
Very high (n=272)	1 (1.4)	3 (1.1)	23 (8.5)	135 (49.6)	110 (40.4)	
TOTAL (n=6177)	69	225	1051	3373	1459	
**Self-Perceived Health In Olders: 65-69 years**						
PAL	Very bad n (%)	Bad n (%)	Fair n (%)	Good n (%)	Very Good n (%)	
Inactives (n=248)	19 (7.7)	54 (21.8)	85 (34.3)	80 (32.3)	10 (4.0)	<0.001
Walkers (n=993)	12 (1.2)	73 (7.4)	329 (33.1)	505 (50.9)	74 (7.5)	
Low (n=195)	0 (0.0)	17 (8.7)	45 (23.1)	117 (60.0)	16 (8.2)	
Medium (n=195)	0 (0.0)	3 (1.5)	48 (24.6)	112 (57.4)	32 (16.4)	
High (n=52)	0 (0.0)	2 (3.8)	11 (21.2)	26 (50.0)	13 (25.0)	
Very high (n=28)	0 (0.0)	0 (0.0)	6 (21.4)	12 (42.9)	10 (35.7)	
TOTAL (n=1711)	31	149	524	852	155	

n: participants; %: percentage; absolute and relative frequency data shown for individuals in age groups for each level of physical activity; PAI: Physical Activity Index; Inactive: PAI=0; reports not walking at least one day a week for more than 10 minutes at a time; Walkers PAI=0; reports walking at least one day a week for more than 10 minutes at a time; Low: PAI=1-15; Medium: PAI=16-30; High: PAI=31-45; Very high: PAI>45; p: p-value from Chi-square test.
